# Magnetic Tunnel Junction as an On-Chip Temperature Sensor

**DOI:** 10.1038/s41598-017-11476-7

**Published:** 2017-09-18

**Authors:** Abhronil Sengupta, Chamika Mihiranga Liyanagedera, Byunghoo Jung, Kaushik Roy

**Affiliations:** Purdue University, School of Electrical & Computer Engineering, West Lafayette IN, 47907 USA

## Abstract

Temperature sensors are becoming an increasingly important component in System-on-Chip (SoC) designs with increasing transistor scaling, power density and associated heating effects. This work explores a compact nanoelectronic temperature sensor based on a Magnetic Tunnel Junction (MTJ) structure. The MTJ switches probabilistically depending on the operating temperature in the presence of thermal noise. Performance evaluation of the proposed MTJ temperature sensor, based on experimentally measured device parameters, reveals that the sensor is able to achieve a conversion rate of 2.5*K* samples/s with energy consumption of 8.8 *nJ* per conversion (1–2 orders of magnitude lower than state-of-the-art CMOS sensors) for a linear sensing regime of 200–400 *K*.

## Introduction

Due to continued device scaling and consequent addition of more components on-chip, which in-turn results in enhanced heat generation, chip temperature monitoring has become a critical issue for ensuring reliable operation. With advanced technology nodes, increased throughput is achieved at the expense of more heat generation. Hence, designing on-chip low-power, low-cost temperature sensors is becoming a crucial requirement^1–4^. The typical performance metrics for on-chip temperature sensors are the conversion rate and energy consumption per inference. The conversion rate is defined as the number of inference samples that can be produced by the sensor per unit sec which is the inverse of the time required by the sensor to make an inference. The energy consumption per inference is defined as the product of the power consumption of the sensor and the inverse of the conversion rate.

While most of the recent work in the domain of on-chip temperature sensors have been primarily based on CMOS sensors^1–4^, it is interesting to note that post-CMOS technologies like spintronic devices demonstrate temperature-dependent probabilistic switching due to thermal noise. Although, traditionally the stochastic switching behavior of spin-based devices have been primarily viewed as a disadvantage for on-chip memory applications, recently unconventional computing paradigms like neuromorphic computing^5–7^, Ising computing^8,9^ and Bayesian inference networks based on stochastic nanomagnets have been proposed that leverage the underlying stochastic device physics. The probabilistic switching of the spintronic device is a function of the input programming current and the operating temperature (assuming a fixed duration of the programming current). However, all these applications abstract the probabilistic switching characteristics of the spintronic device as a function of input current as the external stimulus, at a fixed temperature. This work attempts to explore the stochastic magnet dynamics as a function of temperature and provides an estimation of its performance metrics as an on-chip temperature sensor in comparison to state-of-the-art CMOS based sensors. The potential advantages of such nanomagnetic temperature sensors are compactness, higher conversion rate and lower energy consumption per inference.

## MTJ as Temperature-Biased Random Number Generator

**Figure 1:**
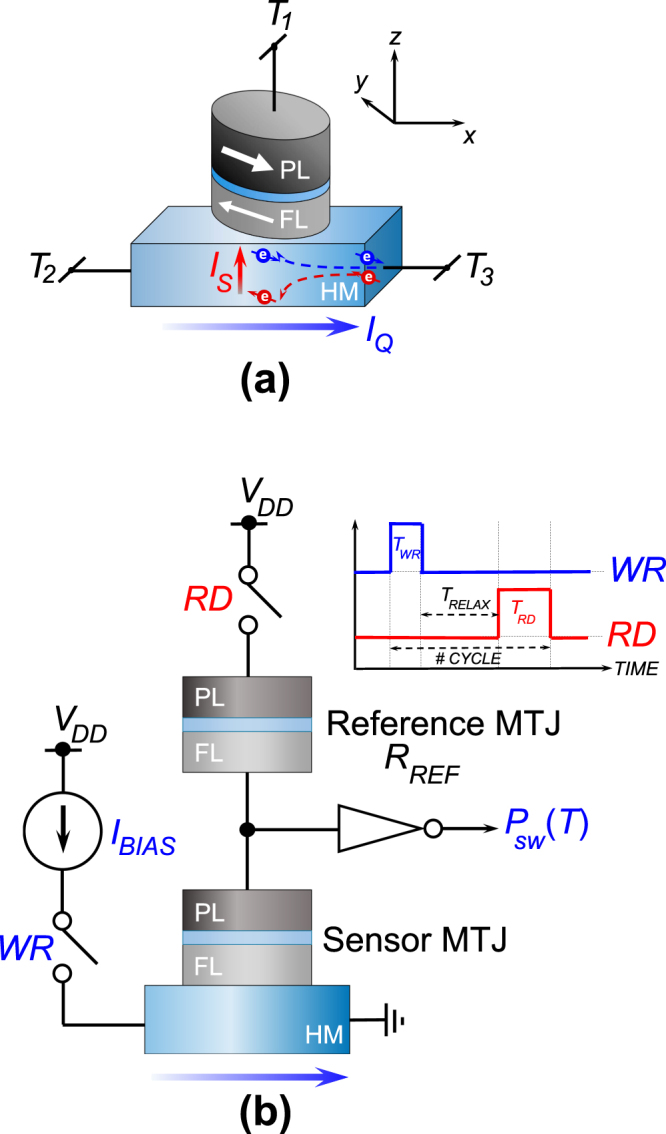
(**a**) The Magnetic Tunnel Junction (MTJ: “free” layer, FL, separated from “pinned” layer, PL, by a tunneling oxide) based temperature sensor is a three-terminal device structure where a charge current, *I*_*Q*_, flowing between terminals *T*_2_ and *T*_3_ of a heavy metal (HM) underlayer results in the injection of an in-plane polarized spin current, *I*_*S*_, on the FL lying on top. “Read” current flowing between terminals *T*_1_ and *T*_3_ is used to “read” the device state, (**b**) The Sensor MTJ is interfaced with a Reference MTJ (*R*_*REF*_) to form a voltage divider circuit (driven by supply voltage *V*_*DD*_) that drives an inverter at the output to determine the switching probability (*P*_*SW*_) at an operating temperature *T*. *WR* and *RD* are control signals that activate the “write” and “read” current paths of the MTJ respectively. During the “write” phase (*WR* activated), a bias current (*I*_*BIAS*_) probabilistically switches the magnet depending on the temperature. After a subsequent “relaxation” phase, *T*_*RELAX*_, the “read” phase (*RD* activated) is used to determine the final state of the MTJ due to the corresponding “write” phase.

An MTJ is a magnetic stack where two ferromagnetic layers are separated by a spacer layer, which is typically a tunneling oxide like *MgO* ^10^. The device exhibits two stable resistive states depending on whether the magnetization of the two ferromagnetic layers are in the same (parallel: P orientation) or opposite (anti-parallel: AP orientation) directions. The resistance of the device is higher in the AP state than in the P state. Figure 1(a) shows an MTJ stack. Note that one of the ferromagnetic layers is denoted as the “pinned” layer (PL) since its magnetization direction is pinned to a particular direction (usually by coupling to an anti-ferromagnetic layer) while the magnetization of the “free” layer (FL) can be manipulated. The MTJ state can be changed from the P to the AP state by passing current through the MTJ from the “pinned” layer to the “free” layer and vice versa due to spin-transfer torque effect^11^. Recent experiments on ferromagnet-heavy metal (FM-HM) bilayers have revealed an alternative energy-efficient mechanism of magnetization reversal due to spin-orbit torque^12–15^. As shown in Fig. 1(a), flow of charge current in the *x*-direction through the heavy-metal layer results in *y*-axis polarized spins to be injected on the ferromagnet lying on top of the HM layer^16^. This input in-plane polarized spin current in the *z* direction can be utilized to switch the ferromagnetic layer of an MTJ with in-plane magnetic anisotropy. The energy-efficiency of spin-orbit torque driven magnetization switching stems from the fact that input electrons scatter repeatedly at the magnet-heavy metal interface, thereby transferring multiple units of spin-angular momentum to the magnet lying on top. The input spin current density (*J*_*S*_) is related to the charge current density (*J*_*Q*_) flowing through the HM underlayer by the relationship, *J*_*S*_ = *θ*_*SH*_.*J*_*Q*_ ⇒ *I*_*S*_ = *θ*_*SH*_.$(\frac{{A}_{MTJ}}{{A}_{HM}})$ *I*_*Q*_, where *I*_*S*_ and *I*_*Q*_ are the input spin current and charge current magnitudes, *θ*_*SH*_ is the spin-Hall angle^16^ and, *A*_*MTJ*_ and *A*_*HM*_ are the MTJ and HM cross-sectional areas, respectively. In this work, we propose spin-orbit torque driven MTJ switching in magnet-heavy metal heterostructures as the underlying physical phenomena for realizing an on-chip temperature sensor. The device structure is depicted in Fig. 1(a), where “write” current flowing through the heavy metal between terminals *T*_2_ and *T*_3_ programs the device state. Subsequently, the MTJ state is “read” between terminals *T*_1_ and *T*_3_.

At non-zero temperatures, the MTJ switching phenomena is stochastic and the degree of stochasticity is governed by the operating temperature. The probabilistic switching characteristics of the MTJ can be analyzed by Landau-Lifshitz-Gilbert (LLG) equation with additional term to account for spin-orbit torque generated by the HM underlayer,

1 $$\frac{d\hat{m}}{dt}=-\gamma (\hat{m}\,\times {{\bf{\text{H}}}}_{eff})+\alpha (\hat{m}\times \frac{d\hat{m}}{dt})+\frac{1}{q{N}_{s}}(\hat{m}\,\times {{\bf{\text{I}}}}_{s}\times \hat{m})$$

where, $\hat{m}$ is the unit vector of FL magnetization, $\gamma =\frac{2{\mu }_{B}{\mu }_{0}}{\hslash }$ is the gyromagnetic ratio for electron, *α* is Gilbert’ s damping ratio, ***H***_*eff*_ is the effective magnetic field including the shape anisotropy field for elliptic disks, ${N}_{s}=\frac{{M}_{s}V}{{\mu }_{B}}$ is the number of spins in free layer of volume *V* (*M*_*S*_ is saturation magnetization and *μ*_*B*_ is Bohr magneton), and ***I***_*S*_ is the spin current generated by the HM underlayer. Thermal noise is included by an additional thermal field^17^, ${{\bf{H}}}_{thermal}=\sqrt{\frac{\alpha }{1+{\alpha }^{2}}\frac{2{k}_{B}T}{\gamma {\mu }_{0}{M}_{s}V{\delta }_{t}}}{G}_{\mathrm{0,1}}$, where *G*_0,1_ is a Gaussian distribution with zero mean and unit standard deviation, *k*_*B*_ is Boltzmann constant, *T* is the temperature and *δ*_*t*_ is the simulation time-step. The first term represents the precession torque, the second term represents the damping-like torque while the third term denotes the spin-torque term. Interested readers are referred to Ref.^18^ for a detailed derivation of the thermal noise term.

The operation of the device as a temperature-biased random number generator has been explained in Fig. 1(b). A particular temperature inference takes place over a number of “write”-“read”-“reset” cycles. The timing waveform for a particular cycle has been shown in the figure. During the “write” cycle, the MTJ is driven by a current source which passes an input charge current through the heavy metal underlayer. Depending on the operating temperature, the MTJ switches with a given probability. Consecutively, during the “read” phase, the MTJ state is determined using the resistive divider circuit shown in Fig. 1(b). The reference resistor, *R*_*REF*_, is an MTJ whose state is fixed in the AP state. The read current is maintained to sufficiently low values such that the MTJ states are not disturbed. Note that the “write” and “read” phases are separated by a “relaxation” period, *T*_*RELAX*_, in order to stabilize the magnetization directions to either of the two stable states after the “write” phase. The magnet is “reset” to the initial AP state for the next cycle in case a switching event takes place by passing a large enough magnitude of current through the heavy metal in the opposite direction to ensure approximately deterministic switching. The switching probability is determined from multiple such measurement cycles and the operating temperature is determined from the measured switching probability.

**Figure 2:**
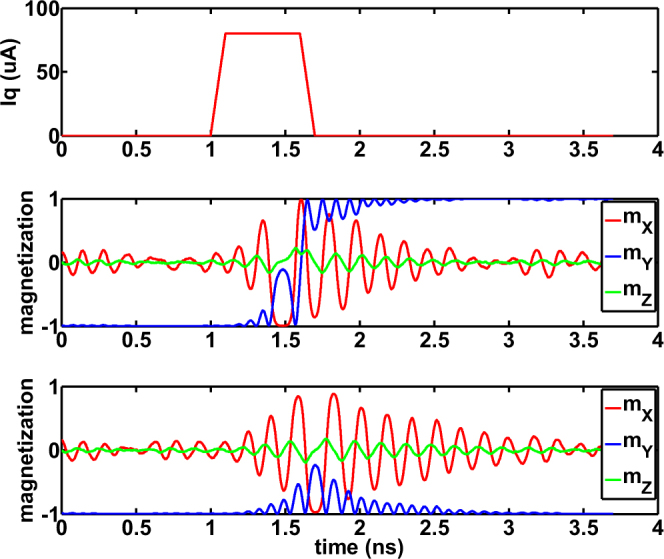
An input current magnitude of $80\,\mu A$ and duration $0.5\,ns$ is passed through the HM layer of the MTJ. Two independent stochastic LLG simulations of the MTJ are shown. The MTJ structure is an elliptic disk of volume $\frac{\pi }{4}\times 100\times 40\times 1.2\,n{m}^{3}$ with saturation magnetization of ${M}_{s}=1000\,KA/m$ and damping factor, $\alpha \,=\,0.0122$. While the MTJ switches in one instance, switching does not occur in the other instance. ${m}_{X}$, ${m}_{Y}$ and m_*Z*_ are the $X$, $Y$ and $Z$ components of magnetization respectively where ${m}_{Y}$ is the magnetization component being switched. Please refer to Fig. 1 for axes directions.

The device parameters have been mentioned in Table 1. The parameters are based on experimental measurements reported in Ref.^13^. Figure 2 depicts two typical independent temporal profiles of the stochastic MTJ magnetization dynamics when subjected to an input current stimulus of magnitude 80 *μA* and duration 0.5 *ns*. While the MTJ switches in the first instance, it stabilizes to the initial state during the “relaxation” period in the other instance.

**Table 1:** MTJ Device Parameters.

**Parameters**	**Value**
Free Layer Area	$\frac{\pi }{4}\times 100\times 40n{m}^{2}$
Free Layer Thickness	$1.2nm$
Heavy-Metal Thickness	$2nm$
Saturation Magnetization, ${M}_{S}$	1000 $KA/m$
Spin-Hall Angle, ${\theta }_{SH}$	0.3^13^
Gilbert’s Damping Factor, $\alpha$	0.0122^13^
Energy Barrier, ${E}_{B}$	20 ${k}_{B}T$ at $T\,=\,300K$
$MgO$ Thickness	$2nm$
Resistivity of HM	200 $\mu \Omega \mathrm{.}cm$
“Write” Phase Duration, ${T}_{WR}$	$0.5ns$
“Relaxation” Duration, ${T}_{RELAX}$	$2ns$
“Read” Phase Duration, ${T}_{RD}$	$1ns$
Design Temperature	$200\mbox{--}400K$

## Sensor Performance Metrics

**Figure 3:**
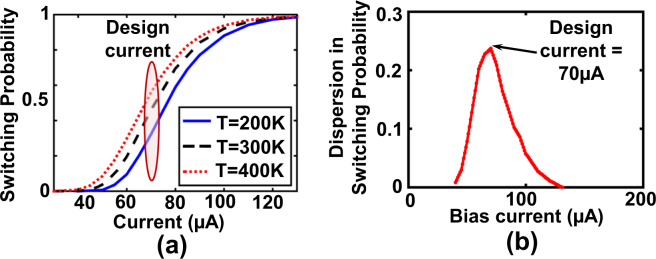
(**a**) MTJ switching probability characteristics with varying temperature in the range 200–400*K*. (**b**) The dispersion in switching probability between $200K$ and $400K$ is maximized for a design bias current $70\,\mu A$ (central region of the switching probability characteristics).

Figure 3(a) represents the switching probability characteristics of the MTJ (as a function of “write” current through the HM) with varying temperature. The dispersion in switching probability characteristics between 200 *K* and 400 *K* is maximized at the central region of the switching probability characteristics (Fig. 3(b)). Specifically, we note that for our design pulse width duration of 0.5 *ns*, the optimal design current is ~70 *μA* and the probability dispersion (absolute difference in the MTJ switching probabilities at 200 *K* and 400 *K*) is ~24%.

**Figure 4:**
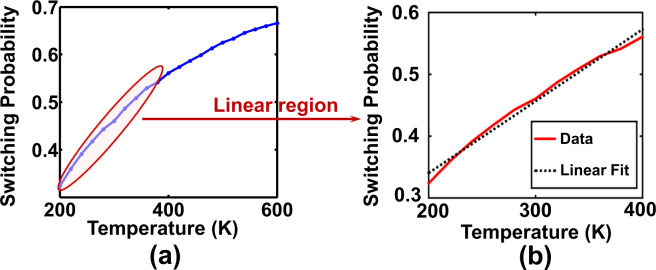
The switching probability of the MTJ subjected to a bias current of magnitude $70\mu A$ and duration $0.5ns$ as a function of temperature. Although the characteristics increase non-linearly, it is approximately linear in the design temperature range of 200–400*K*.

Figure 4 denotes the MTJ switching probability at the optimal bias current of 70 *μA* as a function of temperature. Although the switching characteristic becomes non-linear and tends to saturate at very high temperatures, the characteristic is approximately linear in the range of 200 *K*–400 *K*. The resolution of the sensor linearity is $\sim 0.37 \% /1$  °C.

**Figure 5:**
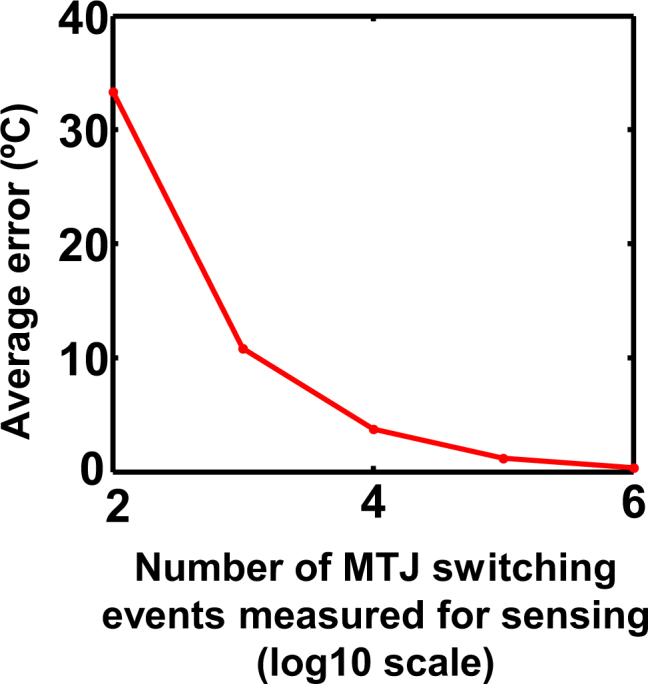
Inaccuracy of the MTJ based temperature sensor as a function of the number of switching events (“write”-“read”-“reset” cycles) used for inferring the switching probability and operating temperature. The average error reduces to $\sim 1$ °C as the number of samples is increased to 100,000.

A single switching event of the MTJ can be considered to be a stochastic process with the probability of switching being determined by the temperature. Consequently, the precision of temperature sensing is expected to increase as the number of switching events (“write”-“read”-“reset” cycles) for the temperature inference process is increased. Figure 5 shows that the average sensing error in the range 200 *K*–400 *K* is reduced to $\sim 1$ °C as the number of samples is increased to $\mathrm{100,000}$. Considering each cycle to be of duration $4\,ns$ ($0.5\,ns$ for “write” phase, $2ns$ for “relaxation” phase, $1\,ns$ for “read” phase and $0.5ns$ for “reset” phase), the resultant time required for one inference is $4\times {10}^{-4}s$ (with an error tolerance of $\sim 1$ °C). The corresponding conversion rate is $2500$ samples/s.

The energy consumption of the MTJ based sensor can be estimated by considering the energy consumed during the “write”, “read” and “reset” phases of operation in one cycle. Considering the bias current of $70\,\mu A$ is provided by a $1V$ supply, the total “write” energy consumption is estimated to be $35fJ$ ($VI{T}_{WR}$ energy consumption, where $V=1V$, $I=70\,\mu A$ and ${T}_{{RESET}}=0.5\,ns$). Assuming a design temperature sensing range of 200*K*–400*K*, the device exhibits a switching probability of ${P}_{RESET}=\mathrm{46 \% }$ at the mean temperature of $300K$. Since, the MTJ needs to be reset for every switching event by passing a $140\,\mu A$ charge current in the opposite direction through the HM layer (to ensure deterministic switching: see Fig. 3(a)), the “reset” energy consumption is estimated to be $\sim 32\,fJ$ (${P}_{RESET}VI{T}_{RESET}$ energy consumption where, $V=1V$, $I=140\mu A$ and ${T}_{RESET}=0.5ns$). The “read” energy consumption was estimated by SPICE simulations of the MTJ based voltage divider driving an inverter stage (as shown in Fig. 1(b)). Non-Equilibrium Green’s Function (NEGF) based transport simulation framework was used to model the MTJ resistance^19^. The total “read” energy consumption was estimated to be $\sim 21fJ$ (including the energy consumption of the latch being driven by the inverter stage). Considering the total number of cycles per inference to be $\mathrm{100,000}$, the total energy consumption of the MTJ based temperature sensor per conversion is given by the product of the resultant energy consumption per cycle and the number of cycles required per inference, and is equivalent to $\sim 8.8nJ$. Comparison of the MTJ based temperature sensor in terms of conversion rate and energy/conversion with other recent proposals of CMOS based temperature sensors are summarized in Table 2.

**Table 2:** Comparison With Other Proposed Temperature Sensors.

Sensor Type	Temperature Range (°C)	Inaccuracy (°C)	Conversion Rate (samples/s)	Energy/Conversion ($nJ$)	Technology
CMOS^1^	$0\mbox{--}100$	$-0.8\mbox{--}+1$	$10$	$150$	$0.35\mu m$
CMOS^2^	$0\mbox{--}100$	$-0.851\sim 0.524$	$1.6K$	$98.13$	$0.18\mu m$
CMOS^3^	$-40\mbox{--}85$	$\pm 1$	$1K$	$66.5$	$0.18\mu m$
CMOS^4^	$20\mbox{--}50$	$\pm 0.1$	$10$	$1600$	$0.18\mu m$
**MTJ (This Work)**	$-73\mbox{--}127$	$\pm 1$	$2.5K$	$8.8$	—

## Scaling to the Super-Paramagnetic Regime

**Figure 6:**
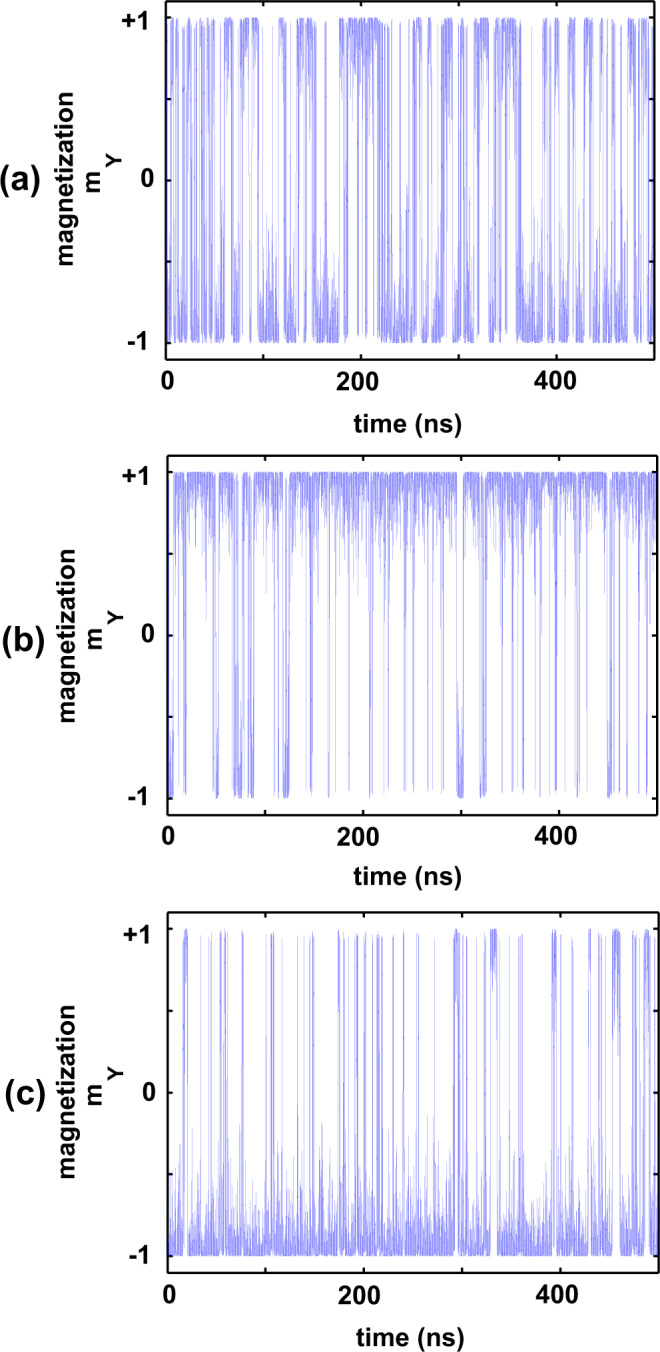
Switching characteristics of a magnet with 1 ${k}_{B}T$ barrier height: (**a**) When the current flowing through the HM layer is zero, the magnet is equally likely to be in the ${m}_{y}=+1$ or ${m}_{y}=-1$ magnetization state, (**b**) When $1.5\mu A$ is flowing through the HM layer, the magnet is more likely to be in the ${m}_{y}=+1$ state, (**c**) When $-1.5\mu A$ is flowing through the HM layer, the MTJ is more likely to be in the ${m}_{y}=-1$ state.

**Figure 7:**
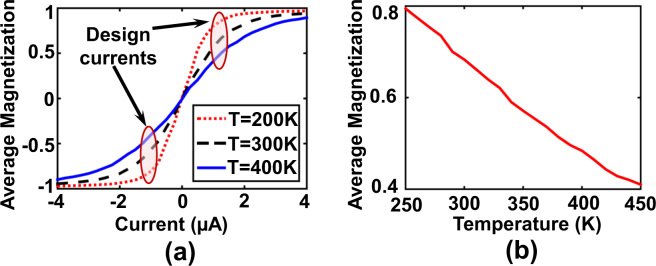
(**a**) Variation of the average in-plane magnetization with magnitude of the “write” current for $T\mathrm{=200}K-400K$, (**b**) For a design bias current of $1\mu A$, the average magnetization varies approximately linearly with the operating temperature. The time-window used for the averaging operation is $\mathrm{100,000}\,ns$.

The discussion so far has been based on magnet dimensions exhibiting a barrier height of $\sim 20{k}_{B}T$ (at the nominal temperature $T=300\,K$). However, as the magnet dimensions are aggressively scaled down to the super-paramagnetic regime ($1{k}_{B}T$ barrier height), the magnet exhibits random telegraphic switching between the two extreme states. As shown in Fig. 6(a), the average dwell time in each state is $\sim \mathrm{50 \% }$, and the average in-plane magnetization over a duration of $500ns$ is approximately zero. The dwell time in either of the two extreme states can be biased by the magnitude of the input current stimulus (flowing through the underlying HM layer) as well as the operating temperature. Figure 6(b,c) depicts the temporal dynamics of the magnetization under the influence of an external current stimulus of magnitude $\pm 1.5\mu A$ at $T=300K$. The average in-plane magnetization is clearly impacted by magnitude of the external current stimulus. Figure 7(a) represents the average in-plane magnetization as a function of the “write” current flowing through the HM layer at the nominal temperature $T=300K$. For a design bias current of $1\mu A$, the MTJ exhibits linear variation of average magnetization profile with sensing temperature (Fig. 7(b)).

Due to the low barrier height, the magnet essentially operates as a volatile device. Consequently, the circuit peripherals have to be operated in an asynchronous fashion (in contrast to the synchronous “write”-“read”-“reset” mode of operation discussed for high barrier height magnets). The “write” and “read” current paths have to be activated simultaneously and the “read” circuit has to be optimized to ensure that the “read” current has minimal impact on the switching of the magnet. Circuit-level simulations indicate that the “read” current can be maintained to values below $100nA$, thereby having negligible influence on the switching probability characteristics of the magnet.

The potential benefits of such super-paramagnetic sensors lies in the conversion rate and energy consumption per inference. Since telegraphic switching occurs in the $\sim ps$ time scale, the time window per inference can be greatly reduced. Further, the “write” bias current magnitude is reduced by almost an order of magnitude, thereby reducing the “write” power consumption. Additionally, no “reset” operation is required (due to telegraphic magnet switching), leading to reduction in both the power consumption and the delay involved in the “reset” operation.

## Conclusions

In conclusion, we proposed a compact nanoelectronic temperature sensor that is able to provide a higher throughput and lower energy consumption in comparison to state-of-the-art CMOS temperature sensors. A key point that enables the usage of stochastic switching behavior of MTJs for temperature sensing applications (in comparison to stochastic switching behavior of other resistive memory technologies) is that the causal element for the device stochasticity is thermal noise. Instead of considering the underlying device stochasticity to be disadvantageous, this work can potentially pave the way for MTJ-enabled on-chip temperature sensors that exploit the probabilistic switching characteristics of nanomagnets at non-zero temperatures.
